# Cardiac autonomic responses after resistance exercise in treated hypertensive subjects

**DOI:** 10.3389/fphys.2015.00258

**Published:** 2015-09-16

**Authors:** Gabriela A. Trevizani, Tiago Peçanha, Olivassé Nasario-Junior, Jeferson M. Vianna, Lilian P. Silva, Jurandir Nadal

**Affiliations:** ^1^Biomedical Engineering Program COPPE, Universidade Federal do Rio de JaneiroRio de Janeiro, Brazil; ^2^Exercise Hemodynamic Laboratory, School of Physical Education and Sport, Universidade de São PauloSão Paulo, Brazil; ^3^Faculty of Physical Education and Sports, Universidade Federal de Juiz de ForaJuiz de Fora, Brazil; ^4^Faculty of Physiotheraphy, Universidade Federal de Juiz de ForaJuiz de Fora, Brazil

**Keywords:** heart rate variability, autonomic nervous system, parasympathetic activity, sympathetic activity, resistance exercise

## Abstract

The aim of this study was to assess and to compare heart rate variability (HRV) after resistance exercise (RE) in treated hypertensive and normotensive subjects. Nine hypertensive men [HT: 58.0 ± 7.7 years, systolic blood pressure (SBP) = 133.6 ± 6.5 mmHg, diastolic blood pressure (DBP) = 87.3 ± 8.1 mmHg; under antihypertensive treatment] and 11 normotensive men (NT: 57.1 ± 6.0 years, SBP = 127 ± 8.5 mmHg, DBP = 82.7 ± 5.5 mmHg) performed a single session of RE (2 sets of 15–20 repetitions, 50% of 1 RM, 120 s interval between sets/exercise) for the following exercises: leg extension, leg press, leg curl, bench press, seated row, triceps push-down, seated calf flexion, seated arm curl. HRV was assessed at resting and during 10 min of recovery period by calculating time (SDNN, RMSSD, pNN50) and frequency domain (LF, HF, LF/HF) indices. Mean values of HRV indices were reduced in the post-exercise period compared to the resting period (HT: lnHF: 4.7 ± 1.4 vs. 2.4 ± 1.2 ms^2^; NT: lnHF: 4.8 ± 1.5 vs. 2.2 ± 1.1 ms^2^, *p* < 0.01). However, there was no group vs. time interaction in this response (*p* = 0.8). The results indicate that HRV is equally suppressed after RE in normotensive and hypertensive individuals. These findings suggest that a single session of RE does not bring additional cardiac autonomic stress to treated hypertensive subjects.

## Introduction

Resistance exercise (RE) has been widely used as an adjunct to aerobic exercise in a comprehensive exercise training program oriented to health (Williams et al., 2007; Garber et al., 2011). Several studies have demonstrated the benefits of RE for increasing muscle mass, strength, balance, and quality of life in older adults or other frail populations (Cheema et al., 2014; Joshua et al., 2014; Silva-Batista et al., 2014; Vechin et al., 2015) and more recent evidence also indicate positive effects of RE in cardiovascular function and regulation (Queiroz et al., 2010; Grizzo Cucato et al., 2011).

Despite these recommendations, a single session of RE promotes a great cardiac autonomic stress, characterized by a reduction in cardiac vagal modulation and an increase in sympathetic activity that persist during the post-exercise period (Heffernan et al., 2006; Rezk et al., 2006; Kingsley and Figueroa, 2014). This autonomic stress promoted by RE has been claimed to be greater than that promoted by aerobic exercise (Heffernan et al., 2006), a fact that can acutely increase the risks of cardiovascular events after RE, particularly in subjects with cardiovascular diseases, such as hypertension (Thompson et al., 2007).

Hypertension (HTN) is a highly prevalent chronic disease (Mozaffarian et al., 2015), characterized by increased levels of blood pressure, end-organ damage, and increased cardiovascular risks (Chobanian et al., 2003). Autonomic dysfunction is one of the main pathophysiological mechanisms of HTN, since it is related both to the development and also the complications of this disease (Mancia and Grassi, 2014). The autonomic dysfunction of HTN has been mainly demonstrated in rest, either by increased levels of sympathetic nerve firing (Schlaich et al., 2004) or reduced heart rate variability (HRV; Singh et al., 1998), but also during physiological maneuvres, such as exercise (Rondon et al., 2006). Accordingly, some recent studies have demonstrated a slower autonomic recovery after aerobic exercise in hypertensives in comparison with normotensives (Erdogan et al., 2011; Aneni et al., 2014).

Given that a single session of RE promotes significant autonomic disturbances, and that autonomic recovery after aerobic exercise is suggested to be slower in hypertensives compared with normotensives, it seems reasonable to expect that autonomic recovery from RE will be further slowed in hypertensives in comparison with normotensives. Thus, the aim of this study was to assess and to compare the cardiac autonomic recovery, assessed by HRV, after RE in treated hypertensive and normotensive subjects.

## Methods

### Sample

Eleven normotensive (NT) and nine hypertensive men (HT) under regular antihypertensive treatment (period of treatment = 9.7 ± 4.9 years; Table 1) participated in this study. Inclusion criteria were: age greater than 50 years old, no smoking for at least 1 year, no practicing of regular physical exercise (frequency up to one session per week) for at least 1 year. None of the study subjects had a history of musculoskeletal injury or cardiovascular diseases that could affect results, no arrhythmias were detected in the resting electrocardiography and none were using beta blockers. All of the subjects provided written voluntary informed consent, which was approved by the University Human Ethics Review Board and followed the recommendations from the Declaration of Helsinki.

**Table 1:** **Antihypertensive medication**.

**Pharmacological class**	**Number of volunteers**	**Percentage (%)**
1. Not on medication	1	11.1
2. Under medication use	8	88.9
2.1. Monotherapy	5	55.6
Diuretics	1	11.1
Calcium channel blockers	1	11.1
Angiotensin receptor antagonist	3	33.3
2.2. Combination of therapies	3	33.3
Angiotensin receptor antagonist and diuretics	1	11.1
Angiotensin receptor antagonist and calcium channel blockers	1	11.1
Angiotensin receptor antagonist, calcium channel blockers and diuretics	1	11.1

### Preliminary assessment

Preliminary evaluations were performed on non-consecutive days. On the first day, the volunteers performed a clinical evaluation, anamneses (personal data, lifestyle questionnaires, previous diseases history, and cardiovascular risk parameters) and physical measurements were taken, such as height and body mass for subsequent calculation of the body mass index (BMI = mass/height^2^; kg/m^2^), waist and hip circumferences for the calculation of waist–hip ratio and percentage of body fat. Single measurements of systolic (SBP) and diastolic (DBP) blood pressures were taken by an experienced evaluator using a calibrated sphygmomanometer after 10 and 20 min of supine resting. The average of these two measurements was retained for analysis. Additionally, mean blood pressure (MBP) was calculated through the sum of the DBP and one-third of the pulse pressure. From the second to the fourth day, the subjects underwent three RE sessions to become familiar with the equipment and exercise techniques. On the fifth and sixth day, individuals performed the test and retest of one-repetition maximum (1 RM) to evaluate the maximum dynamic muscle strength. During the 1 RM assessment, participants were allowed to perform up to five attempts to reach the maximal load for each exercise (see experimental protocol), with rest intervals of 5 min between exercises. Differences lower than 5% between tests were accepted and the greatest 1 RM-value was considered for the prescription of exercise sessions.

### Experimental protocol

The experimental protocol consisted of three phases: rest, RE session, and recovery. All participants were advised not to ingest caffeinated or alcoholic drinks, and not to practice vigorous physical activity in the 24 h prior to the experiments. Initially, the volunteers remained seated for 10 min (rest stage). Then, they were submitted to a RE session in which they performed two sets of 20 repetitions at 50% of 1 RM on the following exercises: leg extension, leg press, leg curl, bench press, and seated row; and 15 repetitions at: triceps push-down, seated calf flexion, and seated arm curl. The rest interval between the sets and exercises was set to 2 min. Finally, individuals returned to the seated position, remaining in recovery for 10 min. At rest and during the recovery phases, heart rate (HR) was recorded beat-to-beat (RR-intervals) by an HR monitor (Polar® RS800CX, Kempele, Finland, sampling frequency = 1000 Hz; Nunan et al., 2009).

### Procedures

#### Heart rate measures

The series of RR-intervals (RRi) recorded during rest and recovery were directed to a microcomputer, by infrared transmission to the Polar Precision Performance software (Polar Inc., Kempele, Finland). After a visual inspection, ectopic beats and artifacts were manually corrected by an expert (J.N.). Then, trend component removal of the time series was carried out according to the “*a priori*” smoothing method (Tarvainen et al., 2002), and interpolation using cubic splines at a frequency of 4 Hz was thus applied to extract equally spaced samples, thereby ensuring series of normal RR-intervals (NN). In sequence, the HR average value of the initial 5 min (rest) and HR-values in each 30-s window during the initial 5 min of recovery were calculated.

#### Heart rate variability analysis: first 5 min of recovery

Since the behavior of the RRi signal in the first 5 min of recovery is non-linear (Goldberger et al., 2006), for the analysis of the HRV in such a period the time-varying vagal-related index RMSSD (square root of the mean of the sum of the squares of differences between adjacent normal R–R intervals) was calculated on subsequent 30 s non-overlapped segments (RMSSD30s), as proposed by Goldberger et al. (2006). To smooth out any transient outliers in the RMSSD30s plots, a median filter operation was applied, where each outlier value was replaced with the median of the value as well as the preceding and following values. The first and last values were not median filtered (Goldberger et al., 2006).

#### Heart rate variability analysis: resting and 5–10 min of recovery

The resting and late recovery period (5–10 min) HRV was analyzed according to the HRV Task Force (Task-Force, 1996) in the Kubios software (v 2.0, Biomedical Signal Analysis Group, Department of Applied Physics, University of Kuopio, Finland). The following time domain indices were calculated: the standard deviation of NN (SDNN), the square root of the mean square differences of successive NN (RMSSD) and the ratio between the number of times in which the difference between successive NN presented a duration higher than 50 ms in relation to the total number of NN (pNN50). The SDNN reflects the participation of all the rhythmic components responsible for variability, and is related to the joint action of both branches of the autonomous nervous system for the control of heart rate, whereas the RMSSD and pNN50 reflect the contributions of variations in high frequencies, which are related to the vagal action on the sinoatrial node (Task-Force, 1996). The frequency domain indices were calculated by the power spectral density function (PSD) using the Fast Fourier Transform (FFT; Malik and Camm, 1990; Task-Force, 1996). Prior to this transformation, the time series were detrended (smoothing priors) and resampled to 4 Hz sampling rate using cubic spline interpolation. For the spectral analysis, the following indices were calculated: the power of the spectral bands of low frequencies (LF; 0.04–0.15 Hz) in absolute units (ms^2^), which represents the set of sympathetic and vagal influences on the sinoatrial node, and in normalized units (nu), which predominantly represents the cardiac sympathetic modulation (Task-Force, 1996); the power of the spectral bands of high frequencies (HF; 0.15–0.4 Hz) in absolute (ms^2^) and normalized (nu) units, which represents the cardiac vagal modulation (Task-Force, 1996); and the LF/HF ratio, whose value is interpreted as a sympathetic-vagal balance indicator (Pagani et al., 1986; Task-Force, 1996).

### Statistical analysis

The results of this study are reported as mean ± standard deviation and the Alpha level was set at 5%. Following the use of the Shapiro–Wilk test, the hypothesis of normality was rejected for SDNN, RMSSD30s, RMSSD, HF, and LF indices, so variables were natural log-transformed (ln). The Student-*t*-test for independent samples and the Mann–Whitney tests were used to compare demographic, anthropometric and haemodynamic variables, and the measures of maximum dynamic muscle strength between the groups. A Two-Way ANOVA (group vs. time), followed by Tukey's *post hoc*-test, were employed to compare the HRV variables between the groups.

## Results

Table 2 presents the demographic, anthropometric, and haemodynamic characteristics of the experimental groups. There were no differences between groups in any of these variables. There were also no differences in maximum dynamic muscle strength in each exercise between groups (Table 3).

**Table 2:** **Sample characterization: demographic, anthropometric, and haemodynamic variables**.

	**HT**	**NT**	***p*-value**
**DEMOGRAPHIC VARIABLES**			
Age (years)	58.0 ± 7.7	56.5 ± 6.3	0.65
	(50–65 years)	(50–74 years)	
**ANTHROPOMETRIC VARIABLES**			
BMI (kg/m^2^)	29.0 ± 3.9	24.8 ± 3.5	0.06
Waist–hip ratio	0.93 ± 0.1	0.88 ± 0.1	0.08
% BF	26.8 ± 7.0	22.7 ± 6.0	0.27
**HAEMODYNAMIC VARIABLES**			
SBP (mmHg)	133.6 ± 6.5	127.0 ± 8.5	0.09
DBP (mmHg)	87.3 ± 8.1	82.7 ± 5.5	0.10
MBP (mmHg)	102.8 ± 6.9	96.2 ± 7.8	0.08

*Values described as mean ± standard deviation*.

*HT, hypertensive group; NT, normotensive group; BMI, body mass index; % BF, percentage of body fat; SBP, systolic blood pressure; DBP, diastolic blood pressure; MBP, mean blood pressure*.

**Table 3:** **Measures of maximum dynamic muscle strength for each exercise**.

**Exercises**	**Maximum dynamic muscle strength (kg)**		
	**HT**	**NT**	***p*-value**
Leg extension	127.7±41.2	130.9±33.8	0.72
Leg press	122.2±37.0	148.4±39.7	0.56
Leg curl	78.4±29.2	92.2±18.3	0.28
Bench press	52.4±15.7	59.6±12.8	0.16
Seated row	99.1±18.4	102.1±16.6	0.75
Triceps push-down	51.0±14.0	54.6±10.1	0.71
Seated calf flexion	34.7±9.3	37.5±5.8	0.43
Seated arm curl	50.0±10.0	55.0±7.8	0.30

*Values described as mean ± standard deviation*.

*HT, Hypertensive group; NT, Normotensive group*.

**Figure 1:**
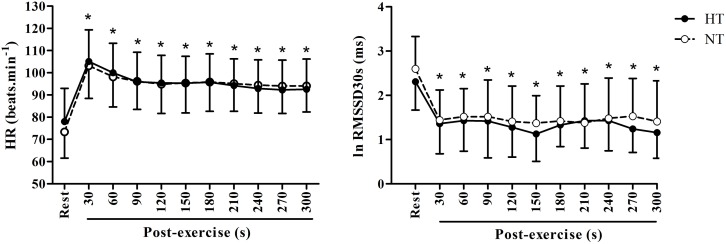
**Heart rate (HR) and heart rate variability (RMSSD30s index) at rest and during 5 min of recovery (post-exercise)**. HT, hypertensive group; NT, normotensive group; ^*^ significantly different from rest in both groups (*p* < 0.05).

The HR and RMSSD30s values in the post-exercise period were, respectively, increased and decreased in comparison to their resting values in both groups (time effect: *p* < 0.01 for all analyses), however, there were no differences in these responses between NT and HT (group vs. time interactions: *p* = 0.10 and 0.83, for HR and RMSSD30s, respectively; Figure 1).

**Figure 2:**
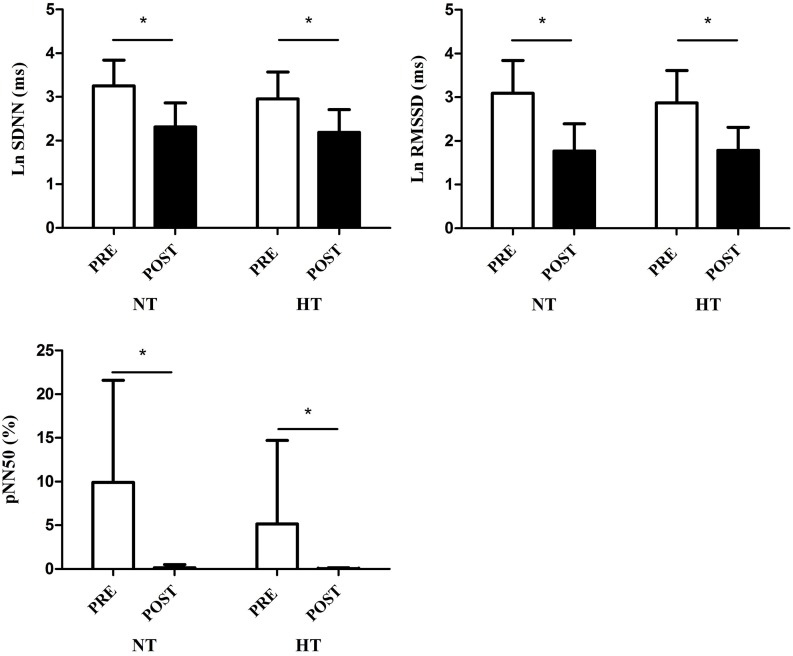
**Time-domain heart rate variability indices (SDNN, RMSSD, and pNN50) at rest (PRE) and during 5–10 min of recovery (POST)**. HT, hypertensive group; NT, normotensive group; ^*^ *p* < 0.05.

**Figure 3:**
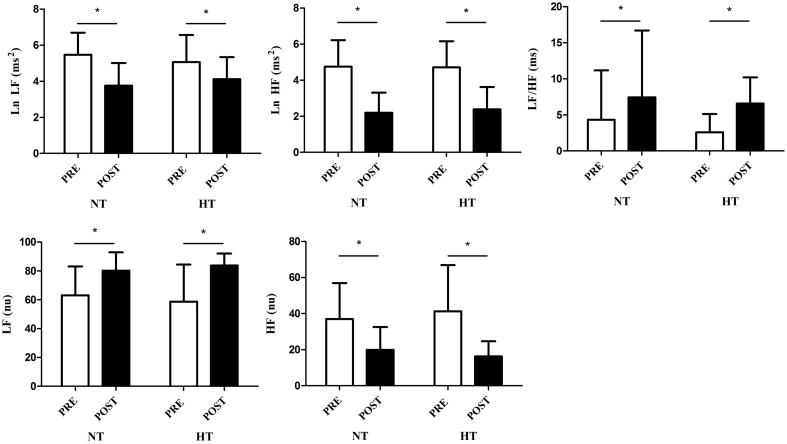
**Frequency-domain heart rate variability indices [LF, HF (ms^2^ and nu) and LF/HF] at rest (PRE) and during 5–10 min of recovery (POST)**. HT, hypertensive group; NT, normotensive group. ^*^ *p* < 0.05.

The mean values of the HRV time-domain indices were reduced in the post-exercise period compared to the resting period in both groups (time effect: *p* < 0.01 for all analyses), however there was no difference in this response between NT and HT (group vs. time interactions: *p* = 0.2–0.4 depending on the index analyzed; Figure 2). Regarding the frequency domain indices, we observed a significant reduction in the LF (ms^2^) and HF (ms^2^ and nu) and an increase in the LF (nu) and LF/HF in the post-exercise period in comparison to resting values (time effect: *p* < 0.01 for all analyses), with no differences between groups in these responses (group vs. time interactions: *p* = 0.2–0.8 depending on the index analyzed; Figure 3).

## Discussion

The main findings of this study were the suppression of the HRV after RE and the lack of influence of hypertension in this response.

It has been demonstrated that an increase in sympathetic and decrease in parasympathetic activity in the post-exercise period underlies the increased risk of acute cardiovascular events in this period (Smith et al., 2005; Thompson et al., 2007). In this context, several studies have been conducted in order to understand which aspects of the exercise could influence the autonomic responses after exercise. Accordingly, the type of exercise seems to play an important role on the post-exercise autonomic recovery. In this regard, Heffernan et al. (2006) compared the HRV after a session of resistance or aerobic exercise, demonstrating a greater reduction of HRV after the RE. Similar results were found by Niemelä et al. (2008), who observed a delayed autonomic recovery after heavy-resistance exercise in comparison with aerobic and light-resistance exercises. Despite the absence of comparisons between resistance and aerobic exercise, the present results are in line with the previous ones (Heffernan et al., 2006) since a significant reduction was observed in parasympathetic activity (reduction in RMSSD and HF) and an increase in sympathetic balance [increase of LF (nu) and LF/HF], leading to an increase in HR and a suppression of HRV during the entire recovery period after the RE session in both groups. These findings spark an alert for the potential risks that could be brought by the practice of RE in subjects more prone to developing cardiovascular abnormalities after exercise, such as individuals with HTN (Thompson et al., 2007).

Hypertension is a chronic highly prevalent disease that is characterized mainly by increased levels of blood pressure, leading to higher rates of cardiovascular morbidity and mortality (Chobanian et al., 2003). The degenerative process of this disease is mediated by some pathophysiological mechanisms and autonomic dysfunction has been advocated as being a crucial one (Mancia and Grassi, 2014). Indeed, studies have already identified an increased sympathetic drive to the kidneys and muscles (Schlaich et al., 2004), and a reduced HRV (Guzzetti et al., 1988; Pagani and Lucini, 2001; Mancia and Grassi, 2014) in hypertensive individuals and it seems that these responses are even worse in more complicated hypertensive states (Grassi et al., 1998a, 2004). Recently, some studies with aerobic exercise have also demonstrated that this autonomic dysfunction in hypertensive subjects is also present in the post-exercise period (Erdogan et al., 2011; Aneni et al., 2014; Best et al., 2014). Accordingly, Erdogan et al. (2011) have demonstrated a slower heart rate recovery after aerobic exercise in hypertensives in comparison to normotensives, and Aneni et al. (2014) observed that this reduction in heart rate recovery accompanies the progression of HTN.

Given that RE is known to produce high levels of autonomic stress, and that hypertensive subjects are supposed to present autonomic dysfunction in the post-exercise period, the hypothesis of this study was that treated hypertensives would present a reduced HRV in the post-exercise period in comparison with normotensives. Despite that, this study did not observe any influence of HTN status on post-exercise HR or HRV. This finding suggests that the autonomic stress imposed by the RE is not different between normotensive and treated hypertensive subjects.

It should be highlighted that the hypertensive subjects in this study were under medication treatment and well-controlled, a factor that is known to improve autonomic function (Kailasam et al., 1995; Ye et al., 2002). This could have prevented a greater post-exercise autonomic stress after RE in this group. Accordingly, most of studies that have shown the presence of autonomic dysfunction in hypertensives have used never-treated subjects (Rondon et al., 2006; Erdogan et al., 2011) or employed a wash-out period before the exercise intervention (Schlaich et al., 2004). Indeed, in the present study HRV-values showed no differences between treated hypertensives and normotensives even in the resting state (i.e., pre-exercise values), a fact that suggests that they did not present autonomic dysfunction in their baseline. The hypertensive subjects of the present study were also free of associated comorbidities and HTN-related complications, factors which are known to negatively influence autonomic function (Grassi et al., 1998b, 2004). Therefore, it seems likely that at least in well-controlled hypertensive subjects with no additional comorbidities and complications, the autonomic stress posed by RE is similar to that of normotensive ones.

The absence of standardization of the antihypertensive drugs among the hypertensive subjects could be viewed as a limitation of this study. However, the modification of the antihypertensive drugs, particularly in well-controlled hypertensives may be problematic, since it could worsen their blood pressure control, thus hampering the treatment. For this reason, the decision to maintain their original prescribed medication was taken in order to warrant the best treatment for each subject. It should also be emphasized that despite each individual using a specific antihypertensive drug (Table 1), no one was under the use of beta blockers, a class of drugs known to directly influence the autonomic nervous system (Frishman, 2003). Another potential limitation of this study could be the reduced number of subjects. However, a *post hoc* power analysis revealed a power >0.7 in most of the statistical comparisons, indicating that the sample size of the current study was adequate for the analysis performed. Finally, it should be emphasized that HRV provides information regarding the cardiac autonomic modulation rather than cardiac autonomic tone *per se*. This means that the tools of the present study did not allow an assessment of the degree of the sympathetic and parasympathetic drives to the heart, but rather its balanced responses to variations in respiration, blood pressure, and temperature, among other factors (Saul, 1990).

## Conclusion

The results of the present study indicate that RE promotes a significant suppression of HRV after exercise, a fact that can potentially increase the cardiovascular risks of the practice of this exercise. However, this response was similar between normotensives and treated hypertensives, suggesting that a controlled hypertension does not bring additional cardiovascular risks to the practice of RE.

## Conflict of interest statement

The authors declare that the research was conducted in the absence of any commercial or financial relationships that could be construed as a potential conflict of interest.
